# Supplementary material for: Extensive nrDNA Polymorphism in Morus L. and Its Application
Source: Plants (Basel). 2025 Aug 18;14(16):2570. doi: 10.3390/plants14162570 (PMC12389063; doi:10.3390/plants14162570)
Supplement: Supplementary file 1 [file plants-14-02570-s001.zip › supplementary/Supplementary S2.zip!/supplementary 2/S2.6.pdf]

```
1        10        20        30        40        50        60        70        80
KF784897.1 TCGAAACCTGCAAAGCAGAAAGACCAGCGGACCCATTACAACACCCAGGGGTGACCCCAACCCCTGACGCTGAGTGTGTGCG
KF784886.1 TCGAAACCTGCAAAGCAGAAAGACCAGCGGACCCATTACAACACCCAGGGGTGACCCCAACCCCTGACGCTGAGTGTGTGCA

       90       100       110       120       130       140       150       160
KF784897.1 GCCCTGCCTTGCGCCCTCAGCATAAAACGAACCCAGGCGCGGAACGCGTCAAGGAATCATAACGAAACGAGCTCCTGCCGTG
KF784886.1 GCCCTGCCTTGCGCCCTCAGCATAAAACGAACCCAGGCGCGGAACGCGTCAAGGAATCATAACGAAACGAGCTCCTGCCGTG

    170       180       190       200       210       220       230       240
KF784897.1 GCCCCGGGGACGGTGATCGCCGCAGCAGCTGTGTTGTGCTTGGTTAAGTCTAAAATGACTCTCGGCAACGGATATCTCGGCT
KF784886.1 GCCCCGGGGACGGTGATCGCCGCAGCAGCTGTGTTGTGCTTGGTTAAGTCTAAAATGACTCTCGGCAACGGATATCTCGGCT

  250       260       270       280       290       300       310       320
KF784897.1 CTCGCATCGATGAAGAACGTAGCGAAATGCGATACTTGGTGTGAATTGCAGAATCCCGTGAACCATCGAGTCTTTGAACGCA
KF784886.1 CTCGCATCGATGAAGAACGTAGCGAAATGCGATACTTGGTGTGAATTGCAGAATCCCGTGAACCATCGAGTCTTTGAACGCA

330       340       350       360       370       380       390       400       410
KF784897.1 AGTTGCGCCTGAAGCCATCAGGCTGAGGGCACGTCTGCCTGGGCGTCAAACACCGATGCCCCCCCAAATCCCCTCGTCACTC
KF784886.1 AGTTGCGCCTGAAGCCATCAGGCTGAGGGCACGTCTGCCTGGGCGTCAAACACCGATGCCCCCCCAAATCCCCTCGTCACTC

        420       430       440       450       460       470       480       490
KF784897.1 TCCCCTGAGTGCCCGGGGAGTGTGGGGGTCGGATGATGGCCTCCCGTGTCTTGGCTCGCGGTTGGCCCAAAGTCGAGTCCTC
KF784886.1 TCCCCTGAGTGCCCGGGGAGTGTGGGGGTCGGATGATGGCCTCCCGTGTCTTGGCTCGCGGTTGGCCCAAAGTCGAGTCCTC

      500       510       520       530       540       550       560       570
KF784897.1 GGTCACGGTTACCGTGGTGACAGGTGGTTGTCGGTCGCTCGGTACCCCGTCACGTGCGCCGGACACGAATCGAGACTCTCTT
KF784886.1 GGTCACGGTTACCGTGGTGACAGGTGGTTGTCGGTCGCTCGGTACCCCGTCACGTGCGCCGGACACGAATCGAGACTCTCTT

    580       590       600       610
KF784897.1 GATTACCCCAACGCATCCCCGTGTGGGTGCCTCTGAT
KF784886.1 GATTACCCCAACGCATCCCCGTTTGGGTGCCTCTGAT
```
